# Surrogate Wars: The “Best Interest Values” Hierarchy & End-of-Life Conflicts with Surrogate Decision-Makers

**DOI:** 10.1007/s10730-024-09546-1

**Published:** 2024-12-26

**Authors:** Autumn Fiester

**Affiliations:** University of Pennsylvania, 423 Guardian Dr, Philadelphia, PA 19102 USA

**Keywords:** Surrogate Decision Making, Futility, Values-Conflict, End-of-Life, Clinical Ethics Consultation

## Abstract

Conflicts involving end-of-life care between healthcare providers (HCPs) and surrogate decision-makers (SDMs) have received sustained attention for more than a quarter of a century, with early studies demonstrating a frequency of HCP-SDM conflict in ICUs ranging from 32–78% of all admissions (Abbott et al. 2001; Breen et al. 2001; Studdert et al. 2003; Azoulay et al. 2009). More recent studies not only acknowledge the persistence of clinical conflict in end-of-life care (Leland et al. 2017), but they have begun to focus on the ways in which these conflicts escalate to verbal or physical violence in the ICU (Slack et al. 2023; Bass et al. 2024; Berger et al. 2024; Sjöberg et al. 2024). I will argue that part of the explanation for the persistence–and even escalation–of ICU disputes is the incommensurable value systems held by many conflicting HCPs and SDMs. I will argue that a common value system among HCPs can be understood as a “Best Interest Values” (BIV) hierarchy, which I will argue is irreconcilable with the set of “Life-Continuation Values” (LCV) held by a sizable minority of families in the United States. I argue this values-conflict undergirds many ICU disputes. If I am correct that an incommensurable value system underlies many ICU conflicts, then it is not just ineffectual for HCPs to impose their BIV system on LCV families, but also wrong given the American commitment to values pluralism. I conclude that the way to navigate continuous ICU surrogate wars is for BIV-focused healthcare institutions to engage more constructively with LCV stakeholders.

## Introduction

Conflicts involving end-of-life care between healthcare providers (HCPs) and surrogate decision-makers (SDMs) have received sustained attention for more than a quarter of a century, with early studies demonstrating a frequency of HCP-SDM conflict in ICUs ranging from 32–78% of all admissions (Abbott et al. 2001; Breen et al. 2001; Studdert et al. 2003; Azoulay et al. 2009). More recent studies not only acknowledge the persistence of clinical conflict in end-of-life care (Leland et al. 2017), but they have begun to focus on the ways in which these conflicts escalate to verbal or physical violence in the ICU (Slack et al. 2023; Bass et al. 2024; Berger et al. 2024; Sjöberg et al. 2024). Because many of these end-of-life conflicts involve disputes over withdrawing life-sustaining therapy (LST), with HCPs deeming those therapies as “futile” or “inappropriate” (Abbott et al. 2001; Breen et al. 2001; Studdert et al. 2003; Azoulay et al. 2009), one prevalent strategy that has been tried during this period is the implementation of hospital-wide or even state-based futility policies. However, recent scholarship has raised questions about both the fairness and legal efficacy of this approach (Truog & Mitchell 2006; Rosoff 2013; Pope, 2016; Kaps et al. 2021). Eliminating futility conflicts has been labeled an “alluring fantasy” by one research team (Nathanson & Feudtner 2016).

I believe this lack of progress is both explainable and expected when viewed through the lens of the respective and incommensurable value systems held by conflicting HCPs and SDMs, what philosopher John Rawls calls their “conceptions of the good” (Rawls 1999). I will argue that a common value system among HCPs can be understood as a “Best Interest Values” (BIV) hierarchy, which I will argue is irreconcilable with the set of “Life-Continuation Values” (LCV) held by a sizable minority of families in the United States. I argue that it is a BIV hegemony that undergirds futility policies and other efforts (including, at times, ethics consultations) to secure SDM compliance with HCPs’ end-of-life recommendations. But for conflicts that involve values discordance, that approach is doomed to fail. In futility conflicts that involve a threat to families’ deeply held values, SDMs will not readily comply or acquiesce with mandates to withhold or withdraw life sustaining treatment (LST). If I am correct that an incommensurable value system underlies many ICU conflicts, then it is not just ineffectual for HCPs to impose their BIV system on LCV families, but also wrong for the powerful gatekeepers of medical access to force values conformity on weaker stakeholders. I argue that, in our pluralistic society, respect for normative difference requires much more serious engagement with individuals holding the LCV cluster.

After offering a description of the BIV hierarchy and evidence of its stronghold among HCPs, I will contrast BIV with LCV using examples of end-of-life and futility conflicts. I will then make the case that honoring patient’s wishes means honoring the LCV of their SDMs, which requires a radical rethinking of futility policies and their justification, and quite possibly an obligation to accommodate families who hold LCV.

## Advance Directives & the Pervasive Problem of End-of-Life Conflicts

Conflict involving the treatment of patients at the end of life has been described in hyperbolic terms by clinicians and researchers. For example, in an editorial in the high-impact journal *Critical Care Medicine*, the authors write that “conflict is present in our ICUs in epidemic proportions” (Long & Curtis 2014, 461). While one study in the journal *Chest* found that conflict with SDMs occurs in only one third of ICU cases (Wilson et al. 2019), other studies in equally reputable journals have found much higher rates of HCP-SDM conflict. In a study published in *Critical Care Medicine*, Schuster et al. asked pairs of patients’ HCPs and SDMs the following question: “How much disagreement, including conflicts and negative feelings, has there been between you and (this doctor/this family) regarding (your loved one’s/this patient’s) care?” (Schuster et al. 2014, 3). They found that conflict was identified by either the HCP or the SDM in 63% of ICU admissions, with HCPs’ perceiving much lower frequency and intensity of conflicts than SDMs (Schuster et al. 2014). The authors conclude that “conflict between physicians and surrogates is prevalent in ICUs” but that HCPs “underestimate the occurrence of conflict relative to surrogates’ perceptions” (Schuster et al. 2014, 6). Although few studies offered insights regarding the types and causes of ICU HCP-SDM conflict, a still widely cited earlier study focused exclusively on “patients who were considered for the withdrawal or withholding of life-sustaining treatment” (Breen et al. 2001, 283–4). Breen et al. found HCP-SDM conflict in 48% of cases where withdrawal or withholding of LST was requested by the clinical team (Breen et al. 2001). The findings of the Breen et al. study are consistent with data on healthcare ethics consultation (HEC), where intractable HCP-SDM conflicts are often referred. Hauschildt and DeVries found that 63% of HEC involved disputes between SDMs and HCPs and that two thirds of these disagreements were cases in which the HCP wanted to withhold or withdraw LST and the SDM wanted LST to continue (Hauschildt & DeVries 2020).

Part of the backdrop for this widespread HCP-SDM conflict is the dearth of advance care planning (ACP) documentation, such as living wills or advance directives. Only 37% of Americans have some form of documented ACP, and the number is only slightly higher (38%) for patients with chronic illnesses (Potter & Lee 2020). But for my argument about values discord between HCPs and SDMs, there is even more telling data. Of patients with living wills or advance directives, 92.7% desired limited care at the end of life and 96.2% wanted comfort care measures only, while only 1.9% of patients with ACP documentation wanted all possible LST (Silveira, Kim, Langa 2010). In other words, patients who desire LST at the end of life do not complete ACP documentation. Work by Garrido et al. offers a rationale for at least one subpopulation of individuals who desire LST: they found that the higher the theological conservativism of the patient, the higher the value the patient placed on receiving all available LST at the end of life and the lower the rate of completing an advance directive (Garrido et al. 2013). Their research showed that the low rates of ACP were intentional, that is, theologically conservative patients actively resist completing living wills or advance directives because they view ACP as undermining God’s control at the end of life. While conflict between HCPs and SDMs can certainly still occur even in the presence of an advance directive, that conflict is more likely to involve an SDM who is advocating for the LST chosen in the patient’s advanced directive, rather than an HCP advocating for an LST-limiting advanced directive that is being resisted by the patient’s SDM (Morrison et al. 2021; Prigerson et al. 2023). And when SDMs concur with the HCP that the patient’s advanced directive should be overruled and LST discontinued, there is no conflict (Smith et al. 2013; Potter & Lee 2020). The vast majority of HCP-SDM conflict occurs in the absence of formal advance directives, or in instances when the SDM attempts to defend the patient’s pro-LST advance directive. I will argue that there is a profoundly discordant *Weltanschauung* between the HCP and SDM at the root of these disagreements, with the HCP prioritizing Best Interest Values (BIV) and the SDM prioritizing Life-Continuation Values (LCV).

## The Best Interest Values (BIV) of Healthcare Providers

In the early bioethics usage of the phrase “best interests,” the term referred to a standard for surrogate decision-making for adult patients articulated by Beauchamp & Childress that lexically ranked a distant third behind the “pure autonomy” and “substituted judgment” standards (Beauchamp & Childress, 1979). If those first two approaches were not possible, then the SDM was charged with having to “determine the highest net benefit among the available options” with the goal to “maximize benefit through a comparative assessment” (Beauchamp & Childress 1979, 102). The “best interests” standard for surrogate decision-making named a method for determining the right course of action for the patient, but it was agnostic about what constituted a particular patient’s best interests. In fact, the critics of the “best interests” standard argued that it was too vague and ill-defined to be useful because it was pure procedure and no content (Veatch, 1995; Koppleman 1997; Salter 2012). But over the course of the next few decades, the “best interests” method for surrogate decision-making morphed into the “Best Interest Values” (BIV) hierarchy of HCPs. No longer merely demarcating a process of weighing and balancing various health and non-health interests, the BIV became a substantive “conception of the good” with unambiguous, established normative commitments. This problematic because the BIV commitments of HCPs may not only be irrelevant to what the patient or family describes as being in the patient’s “best interest,” but those normative commitments may actually be antithetical to what the patient or family believes is in the patient’s best interest. In an essay on parental decision-making at the end of life, Kingsley et al. raise a similar concern in the pediatric context, arguing that the BIV is “used by clinicians to introduce their own values in the decision-making calculus” and that BIV values “do not account for the differences in how clinicians and families define a child’s best interest” (Kingsley et al. 2023, 3).

Rawls’ “conception of the good” concept is meant to encapsulate an individual’s core values, life priorities, spiritual beliefs, and views about the meaning of life and what is constitutive of a good human life (Rawls 1999). The contemporary paradigm of informed consent purports to prioritize the patient’s value system with the “aim to protect patients’ personal autonomy from paternalistic medical interference” (Childress & Childress 2020, 423). The HCP’s conception of the good and personal healthcare values are not to be imposed on patients because this would thwart the obligation “to respect patients as individuals and to deliver care consistent with *their* values and preferences” (Beach & Sugarman 2019, 811, *italics added*). This amounts to a mandate of HCP “value-neutrality” about which conception of the good is best, yet I will amass a tapestry of evidence below to demonstrate that a widely shared HCP conception of the good plays a significant role in clinical recommendations. Renowned bioethics scholar, Robert Veatch, raised concerns about this in the 1990s, arguing that HCP value-neutrality about conceptions of the good was a mirage despite the rhetoric of patient autonomy and informed consent. He worried that “the consent model” was just the “traditional, authoritarian understanding of clinical decision-making” masquerading as patient choice (Veatch 1995, 6). Veatch is not alone in raising this concern about HCP values imposition in the informed consent process (Barry 1999; Entwistle & Watt 2016; Frosch et al. 2012). Others have flagged the risk of “residual paternalistic tendencies among many clinicians” (Childress & Childress 2020, 424). Undergirding clinical decision-making is a discernible values hierarchy that takes a definitive stance on meaningful existence, the good death, appropriate healthcare consumption, proper utilization of resources, and the rightful place of spirituality in medical care. Though this worldview is an articulation of HCPs’ own normative priorities–and is therefore inherently subjective–it claims value-neutrality because it is cloaked in the language of patients’ own “best interests.” I agree with Salter that “best interest language is appealed to all the time” (Salter 2012, 189) in clinical recommendations. Veatch captures this worry when he claims that “doing what is best for the patient has achieved the status of an unquestionable platitude” (Veatch 1995, 6). It is a “platitude” in the sense that the HCP’s actions, in trying to achieve a patient best’s interest, no longer has to be subject to critical reflection or scrutiny. And because HCPs seem to believe that their clinical actions really do serve patients’ objective best interests, they are blind to the perspectival nature of their values framework, viewing it instead as simply the right course of action to achieve the best outcome for the patient given the objective facts. I use the nomenclature of “Best Interest Values” (BIV) to describe this framework.

To illustrate the core tenets of BIV, I will use a variety of sources to create a tapestry of this conception of the good. Although BIV infuse clinical practice outside of the context of adult end-of-life care, my intention is to distill from these examples the set of normative precepts that anchor HCPs’ positions in intractable futility disputes. The test of the accuracy of this depiction is whether it resonates with HCPs: do the majority of HCPs recognize this normative framework as their own?

The central tenets of the BIV hierarchy can be seen in the case description offered by physician-bioethicist Robert Fine to defend the Texas Advanced Directive Act (TADA) (Texas 2023), which allows Texas hospitals to withdraw LST that they deem “medically inappropriate” regardless of the patient’s wishes or the wishes of the SDM. Fine, who was actively involved in writing the 1999 law (Fine 2009), used this case to argue the merits of TADA in an essay 10 years after its implementation (Fine 2009). He writes, “I believe the law fosters the best interests of patients in extraordinarily difficult cases like that of Mr. J” (Fine 2009, 967). Because the case explicitly defends the position of unilateral withdrawal of LST against the wishes of the patient’s SDMs on grounds of patient’s best interests, it serves as a rich source for illustrating the BIV hierarchy. These normative commitments can be detected in the excerpt using content analysis–that is, not merely through what is literally stated, but through the features of the case that are emphasized and the facts of the case that are deemed essential to include. Fine writes,

Mr. J is an 82-year-old man who has renal failure, congestive heart failure, and advanced dementia. For the past 2 years, he has been bed bound in a nursing home, receiving artificial nutrition and hydration via a gastric tube, and transferring to a dialysis center by ambulance three times a week. He has now been admitted to the acute care hospital for the third time in the past 6 months, this time with respiratory distress secondary to pneumonia and exacerbation of heart failure...The patient is intubated in the ED, started on therapy with pressors and IV antibiotics, and placed in the ICU. One week later, Mr. J is extubated and moved to a hospital room, requiring therapy with pressors intermittently during dialysis. The nursing staff, social workers, and physicians are very concerned about the patient’s suffering and recommend “comfort care only” level of treatment…The children are increasingly perceived as “in denial,” meddlesome, and potentially litigious. A palliative care consult is requested. The palliative care team finds a patient with advanced dementia lying in a semifetal position and displaying multiple nonverbal criteria for pain, including moaning and grimacing. The family expresses a strong belief that the patient is not in pain…They reject suggestions for any pain medication…After a week of multidisciplinary dialogue with the family by the palliative care team, the family continues to say that they expect doctors and nurses to “do everything,” including dialysis, reintubation if needed, and full cardiopulmonary resuscitation, noting that “if it’s God’s will, God will take him”…Our patient cannot recover enough to live free from the hospital, is either suffering or unable to appreciate any joy in being alive, and cannot die easily or peacefully without permission. We conclude that further aggressive treatment is futile. (Fine 2009, 963)

I want to highlight 8 normative commitments of the BIV cluster that can be distilled from this excerpt, all of which are subjective values-claims that conflict with other possible conceptions of the good, including the Life-Continuation Values (LCV) hierarchy that I will discuss below. My argument is that Fine’s BIV represent widely held values among HCPs that result in interminable conflict with SDMs holding opposing values. In addition to signaling the occurrence of these values in this excerpt, I will invoke other evidence of these BIV tenets in the hope of demonstrating how widespread this value system is among HCPs. The tenets of BIV include:

### Nonexistence Can be Preferable to Existence, i.e., Being Dead can be a Better for a Person than Being Alive

Beauchamp and Childress articulate this normative claim in their *Principles of Biomedical Ethics*, claiming, “Suffering and loss of cognitive capacity can ravage and dehumanize patients so severely that death is in their best interests” (Beauchamp & Childress, 1979, 184). The BIV hierarchy considers death to be preferable to living either on grounds of suffering or significant cognitive impairment. Both grounds can be seen in Fine’s inclusion of the statement, “*Our patient cannot recover enough to live free from the hospital, is either suffering or unable to appreciate any joy in being alive*.” The moral distress literature shows parallel beliefs among nurses (Morley and Sankary 2023). One of the most common causes of moral distress is described as: “Follow the family’s insistence to continue aggressive treatment even though I believe it is not in the best interest of the patient” (Epstein et al. 2019, 118). I coined the term “value-negative existence” to describe a HCP’s assessment that a patient’s existence, on balance, entails more pain and suffering than any amount of benefit that can come from its continuation (Fiester 2014). Commenting on a lawsuit involving a patient in a persistent vegetative state, Dr. David Pate, the chief medical officer of the hospital involved, told reporters, "He's unaware of his surroundings, he can't eat, he can't speak, he can't move any of his extremities. I can't imagine anybody in his condition wanting extraordinary means of life support to be kept alive" (Ackerman 2005). That assessment is not limited to patients with minimal cognition. Anthropologist William Peace, paralyzed since youth and hospitalized with a serious infection requiring a long recovery, describes an interaction that conveyed the HCP’s view that his life had become a value-negative existence: “What transpired after the nurse exited the room has haunted me. Paralyzed me with fear…His next words were unforgettable. The choice to receive antibiotics was my decision and mine alone. He informed me I had the right to forgo any medication, including lifesaving antibiotics. If I chose not to continue with the current therapy, I could be made very comfortable. I would feel no pain or discomfort at all” (Peace 2012, 14–15). Peace concluded, “Clearly death was preferable to nursing home care, unemployment, bankruptcy, and a lifetime in bed” (Peace 2012, 15).

### Sustaining Life Through Medical Interventions Is Morally Wrong when the Burdens (Invasive Treatments, Physical Pain, Long Recovery) are not Offset by the Prospect of Meaningful Existence or Medical Benefit

Fine’s patient, Mr. J, is described as having advanced dementia, being bed bound, and lying in a semifetal position. In the BIV hierarchy, this state of existence cannot possibly justify the catalog of Mr. J’s medical burdens that include artificial nutrition and hydration, dialysis, intubation, pressors and IV antibiotics. This burden-benefit balancing (Shea 2020) is central to BIV, where many clinical interventions are understood to be harms or burdens and “medical benefit” is understood as clinical improvement, a return to baseline function, reasonable chances of being weaned off of LST, or restoration of cognitive function – not merely the prolongation of life. This is evident in Fine’s case because the interventions of being “*intubated in the ED, started on therapy with pressors and IV antibiotics, and placed in the ICU”* were successful in prolonging Mr. J’s life since “*[o]ne week later, Mr. J is extubated and moved to a hospital room.”* In the BIV, the burden of invasive therapies is not justified by the mere extension of biological existence. If it were, the case of Mr. J would appear as a resounding medical success story. We are told that Mr. J “*has now been admitted to the acute care hospital for the third time in the past 6 months,”* which means that he has been able to be discharged from the acute care hospital twice before. In their content analysis of ethics consultation case descriptions and recommendations, Hauschildt and DeVries found many instances of this logic and many case summaries similar to their “Case 120”: “Given the irreversible nature of her underlying disease(s), the primary team feels the action involved in a cardiopulmonary resuscitation (chest compressions, shocks, etc.) would provide little to no medical benefit while inflicting significant harm” (Hauschildt and DeVries 2020, 312). The HCPs involved in “Case 120” did not claim that cardiopulmonary resuscitation would fail to extend the patient’s life, but that the higher standard of “medical benefit” would not be met.

### Meaningful Existence is Incompatible with Severe Cognitive Impairment or Significant Intellectual Disability

The BIV system places a high premium on quality of life, and it takes a definitive stance on what constitutes an acceptable quality of life: patients must have significant cognitive awareness. Fine employs a shorthand to describe where the bar is set for sufficient quality of life, writing that Mr. J is “*unable to appreciate any joy in being alive.”* In a recent “Clinical Ethics Case Studies” section of the *Hastings Bioethics Forum*, bioethicists Malek and Nelson describe an ethics conflict involving a 21-year-old man with profound cognitive disability, whose mother wants him to have surgery for a brain mass. Although the surgeons would remove such a mass in a 21-year-old neuro-typical patient, Malek and Nelson report that “one of the physicians says that he thinks that J’s intellectual disability limits his ability to understand and appreciate the value of continued existence, and, therefore, the surgery would not benefit J ‘in any meaningful way’” (Malek & Nelson 2022). Parents in a study of families with children with trisomy 13 and 18, which causes severe intellectual disabilities, reported that “health care providers did not see their baby as having value, as being unique, as being a baby” (Janvier et al. 2012, 296).

### Meaningful Existence is Incompatible with Severe Physical Impairment or Disability

In the BIV system, physical dependence (e.g., paralysis, the inability to complete activities of daily living, being bedbound) is deplorable. Fine’s choice to include the description of Mr. J as *“bed bound”* and *“lying in a semifetal position”* evokes the sense that Mr. J lives in an intolerable state. While Mr. J had both physical and cognitive impairment, William Peace did not, and Peace wrote that “physicians or others involved in treatment decisions did not understand or appreciate the prospects of people with disabilities to live good quality lives” (Peace 2012, 15). A 2021 *Health Affairs* study confirms Peace’s claim: 82.4% of physicians in that study believed that people with spinal cord injuries (SCI) have worse quality of life than nondisabled people, and while only 18% of physicians imagined they would be glad to be alive with a severe SCI, 92% of patients with an actual SCI reported being glad to be alive (Iezzoni et al. 2021). This BIV perspective is clearly discernible in the exchange reported by Melvileen Klein at a TADA hearing of the Texas State Legislature. Klein told the hearing members about the ethics committee meeting she attended to determine whether the hospital would invoke TADA and withdraw her mother’s LST. According to Klein, the physician-chair said, “I think we can come to a conclusion by asking five simple questions. Can your mother feed herself? Can your mother dress herself? Can she stand up? Can she wash herself? Can she use a bedpan?” When Klein indicated that her mother, 91-year-old Edith Lois Pereira, could not perform those tasks, the physician-chair responded, “There, I think we have our answer” (Hearings 2006).

### LST is not Justified for the Purpose of Merely Continuing Unconscious or Minimally Conscious Existence, or Sustaining a Clinical Status Quo

When Fine writes, **“We conclude that further aggressive treatment is futile,”** he does not mean that the interventions “cannot accomplish their intended physiologic goal” (Bosslet 2015, 1318). Each intervention has clearly achieved its physiological purpose, which is why Mr. J has been able to be discharged back to the nursing home twice, and, in the current hospital admission, has been weaned off of the ventilator within a week and moved to a regular medical floor. Feudtner and Nathanson explain the usage of the term “futile” in the BIV values hierarchy: “Stated more bluntly, some treatments are labeled as futile because the resulting quality of life after the intervention is not deemed by the labeler as worthy of the intervention” (Feudtner and Nathanson 2018, 353). Mr. J’s treatment is labeled “futile” because his advanced dementia prevents him from reaching Fine’s bar of being able to “*appreciate any joy in being alive”* and because his “*renal failure, congestive heart failure”* will never improve. This BIV standard for continuing longer-term LST is evident in many of the legal challenges brought against TADA since its enactment. A typical example is the case of nine-month-old Tinslee Lewis, who was born with a heart defect called Ebstein’s anomaly and was intubated and receiving artificial nutrition and hydration when Cook Children’s made the decision to withdraw her LST. After her mother, Trinity Lewis, secured a restraining order, the hospital released a statement defending their decision and providing their rationale. Their statement exemplifies this BIV principle about LST: “In the last several months, it’s become apparent her health will never improve. Despite our best efforts, her condition is irreversible, meaning it will never be cured or eliminated. Without life-sustaining treatment, her condition is fatal…Removing this beautiful child from mechanical ventilation is a gut-wrenching decision for Cook Children’s physicians and staff, however, we feel it is in her best interest to free her from artificial medical intervention and suffering” (WFAA 2019). Having won the legal challenge, Tinslee continued to receive LST and was discharged from the hospital to her home three years later.

### For Incapacitated Patients, Eliminating or Minimizing Physical Pain is an Overriding Clinical Obligation, Even if it Conflicts with other Interests

Fine describes Mr. J as *“displaying multiple nonverbal criteria for pain, including moaning and grimacing,”* but we are told that *“[t]he family expresses a strong belief that the patient is not in pain”* and* “[t]hey reject suggestions for any pain medication.”* It is telling that we do not learn the family’s reasons for resisting pain medicine: in BIV, those reasons are irrelevant. Pain relief trumps other considerations and interests for incompetent patients in the BIV hierarchy. In an end-of-life case study, four intensive care physicians were asked if they would allow the sedation to be lifted briefly for a 57-year-old dying patient to enable his family to briefly interact. The patient had experienced unexpected complications from a surgery that was predicted to have a positive outcome. All four of the clinicians said they would reject the pleas of the family to have a few final minutes with the patient unsedated. One physician summarized the BIV perspective: “The risk of allowing the patient to awaken is unacceptable in the light of the weak (or absent) arguments for possible benefits that might accrue” (Batchelor 2003, 336). Another physician commented, “We will not allow him to be in pain” and another commented, “In such a situation, to date, I have not agreed to stop sedation” (Batchelor 2003, 336).

### Spiritual Beliefs Should Play a Supportive Role for the Family, not a Proscriptive or a Directive Role in the Clinical Treatment of an Incapacitated Patient

While competent patients are free to allow theological commitments to dictate their own treatment priorities (e.g., Jehovah Witness patients’ refusal of blood products), BIV reject the idea that the family’s theological commitments should dictate the clinical course, especially in the decision regarding continuation of LST. Mr. J’s family’s pro-LST argument that *“if it’s God’s will, God will take him”* is given no weight in the decision regarding LST withdrawal. In fact, that statement in the case narrative functions to undermine the credibility of Mr. J’s family, rather than enhance it. In the above study about lifting the sedation for the dying 57-year-old, one of the physicians explains the BIV perspective on spirituality and medical care: “The decision whether to wake this patient for purposes of closure concerns less his medical treatment than his spirituality and, under the facts of this case, can have no bearing on his physical health at all” (Batchelor 2003).

### A Good Death is a Death Outside of the ICU and Free of LST

BIV has a decided view about the best kind of death: death should be intervention-free, preferably in hospice, with the patient receiving comfort care measures only (Hauschildt 2022). Meeting none of these criteria, Fine laments that Mr. J “*cannot die easily or peacefully without permission.”* Traudt et al. have shown similar views among nurses who hold the “conviction that withdrawal of aggressive treatment and support of as peaceful of death as possible was in the best interest of the patient” (Traudt et al. 2016, 206).

I have stitched a tapestry of the BIV hierarchy to show that it is a coherent conception of the good that encompasses specific normative claims which undergird the positions many HCPs take in end-of-life and futility conflicts. HCPs holding BIV are not value-neutral about end-of-life care but rather express a particular understanding of what would be in a patient’s “best interest.” But there is an alternative, competing worldview about what is in a patient’s “best interest” that is also coherent, and it grounds SDMs’ resistance to withholding or withdrawing LST in end-of-life care. I refer to this conception of the good as the “Life-Continuation Values” (LCV) hierarchy.

## The Life-Continuation Values (LCV) of Surrogate Decision-Makers

The BIV cluster stands in stark contrast to the LCV standard held by a sizable minority in the US. This LCV conception of the good lies beneath many of the most well-known SDM brain death or futility conflicts, including those involving Terry Schiavo, Emilio Gonzalez, and Jahi McMath. We describe six tenets of the LCV conception of the good distilled from the testimonials of SDMs, and their supporters, who have been involved in end-of-life and futility disputes.

### Human Life has Intrinsic Worth in any State or Condition, so Being Alive is Always a Good and Being “Alive” Means Having a Heartbeat

Articulating this perspective, Terry Schiavo’s mother, Mary Schindler, said of individuals in a persistent vegetative state like her daughter, “They’re people. They’re a person” (Hannigan 2006). Four years after 11-year-old Jahi McMath was declared brain-dead in California and moved to New Jersey, her mother chaffed at the suggestion that her daughter was “deceased.” She remarked, “It’s the principle: I really have a human being that I get up and see about every day” (Aviv 2018). Regardless of physical or cognitive capabilities, consciousness or brain activity, human beings are never better off dead than alive on the LCV worldview. Terry Schiavo’s father, Bob Schindler, told a reporter after her death that some families of brain-damaged patients say that “it’s better off that they’re dead.” He responded, “Well, we say that’s baloney. Human life is precious” (Hannigan 2006). In fact, the book they co-wrote after her death was titled, *A Life That Matters* (Schindler & Schindler 2006). William Peace remarked on how puzzling that view is to the able-bodied or neuro-typical, commenting, “We admire people with a disability who want to die, and we shake our collective heads in confusion at those who want to live” (Peace 2012, 16). Disability advocate, 27-year-old Drew Clayborn, who has been quadriplegic since high school, echoed this view, saying, “My life matters. It's better that I'm here now than if I had died that day” (Fromson 2022). In the LCV hierarchy, there is no distinction drawn between physical impairment and cognitive impairment: they are both categories of disability, and all disabled individuals require equal protection. Mary Schindler lamented, “There was absolutely no reason for her to die. That’s what I think about. She was disabled” (Hannigan 2006). Commenting in his religious notebook about God’s plan for him, Terry Schiavo’s 12-year-old nephew wrote, “God’s plan for my family is to fight for those that are disabled” (Hannigan 2006). Adherents of the LCV recognize one dichotomy: being alive, which accords full dignity and rights to a person, and being dead, which most will define as cardiac death. Tiffany Hostetter, who fought the TADA withdrawal decision for her nine-year-old daughter, Payton Summons, said, “Her heart rate was still great. Her organs were functioning. She's still alive” (Unger 2018).

### Each Human Life Should be Extended as Long as Possible, with Maximizing the Quantity of Life Being the Ultimate Goal of Medical Interventions

The clinical ethicist in Hauschildt and DeVries’ “Case 120” describes a family holding this exact view: “[The patient’s] family remains firm in their wish that the patient remain a FULL CODE with the expressed hope that this will prolong her life” (Hauschildt and DeVries 2020, 312). Researchers Feudtner and Nathanson explain this LCV perspective: “[I]f the utility of remaining alive even for another moment is infinite, then even a vanishingly small probability of success would combine to make the pursuit of treatment rational” (Feudtner and Nathanson 2018, 353): For people who hold LCV, their loved one’s survival and continued existence is of paramount importance. Like Tiffany Hostetter, Jeannette Nikolouzos got a restraining order against her husband’s Texas hospital when they decided to withdraw his LST. Spiro Nikolouzos had been in a persistent vegetative state for four years. His wife had cared for him at home until his recent hospitalization when he was placed on a ventilator for respiratory distress. After securing a court order to continue his LST, she said, "I can't tell you the relief and excitement I feel that my husband is still alive" (Ackerman 2005). As a result of her legal challenge to the withdrawal of her mother’s LST, Melvileen Klein secured nursing home placement for her mother, Edith Lois Pereira. In her obituary eight months later, Klein thanked “St. Joseph Hospital, where she received the care she needed to survive and enable the family to be with her for 8 additional months” (*Houston Chronicle* 2007). Four years after being declared brain-dead, Jahi McMath experienced cardiac cessation. Her mother made a statement, saying, “She was a little girl who deserved to be cared for and protected” (Chow 2018).

### Quality of Life Judgments Should not be Made to Determine Who Lives or Dies

Individuals with LCV believe the HCPs have no moral authority to make life or death decisions about patients based on HCPs’ view of patients’ quality of life. They see such judgments as standing outside the legitimate purview of clinical expertise. Kimberlyn Schwartz, spokesperson for Texas Right to Life, said, “These are not medical decisions. They’re value judgments on whether someone else’s life is worth living” (CBS Texas Staff 2022). This same view was echoed by Melanie Childers, who secured a restraining order preventing the LST withdrawal for her sister, Andrea Clark. In an interview about the TADA process, she said, “If their ethics committee makes a decision, it doesn’t matter what the patient wants. They just say, ‘Well she’s miserable.’ Well, to me that’s a quality of life decision that is up to her and her family. That is not a medical decision” (Boggs 2006). The National Council on Disability, an independent federal agency that advises the President and Congress, agrees. In their 2019 report, “Medical Futility and Disability Bias,” they argued, “As a matter of principle, only the patient’s value judgments regarding quality of life should matter in futility decisions” (National Council on Disability 2019, 36). After 20 years of permitting quality of life judgments to play a substantial role in LST withdrawal decisions, the Texas state legislature amended TADA in 2023, which now aligns with this LCV perspective. Through bill HB 3162, hospitals’ decisions to withdraw LST must be made “without regard to any judgment on a patient’s quality of life” (HB 3162).

### Minimally Conscious and Unconscious Persons Receive Benefits from Being Alive and are not “Suffering”

LCV adherents do not accept HCPs’ assessments that their loved ones are “suffering” by being alive in this state. While there is a constant refrain from HCPs and hospitals that these patients are “suffering” acutely, individuals who hold LCV do not see anguish, torture, despair, or agony when they interact with their loved ones. They see persons who can receive love, who have an awareness that they are cared for, and who interact meaningfully. Having visited her daily for a decade, Mary Schindler recalled “her smile, the way she laughed, how she knew who I was. The way she tried to talk to me. I think of her every day—just how happy she was… She was brain-damaged and she was a happy, happy little girl” (Hannigan 2006). In an interview after Jahi McMath was buried, her mother Nailah Winkfield told the reporter, “[M]y daughter knew I was there and that I loved her” (Goldschmidt 2018). The mothers who fought TADA decisions all had similar stories. Emilio Gonzalez’s mother, Caterina Gonzalez, said, "I put my finger in his hand, and I'm talking to him, and he'll squeeze it. Then he'll open his eyes and look at me" (Cohen 2007). Tamiko Dismuke, said of her six-month-old daughter, Knya Dismuke-Howard, "She's fully aware. She opens her eyes when we enter the room and makes facial expressions, like moving her eyebrows or pouting” (myplainview.com April 29, 2005). Wanda Hudson, who lost her legal battle and whose son had his ventilator removed in 2005, said of 6-month-old baby, Sun, “I wanted y’all to see my son for yourself, so you could see he was actually moving around. He was conscious” (Hopper, 2005). After three years in Cook Children’s Hospital, Tinslee Lewis, now a three year old, was discharged home with LST. After discharge, her mother, Trinity Lewis, reported “She’s doing so good” (Liou 2022).

## Withdrawing LST is Morally Wrong Because it is a form of Killing

Individuals who hold LCV view LST-removal as the willful, intentional, affirmative act of causing a person’s death. They find no credence to the HCPs’ claim that withdrawing LST merely gives “permission” for the patient to die “peacefully,” to use Fine’s wording. Evelyn Kelly recounted her response to the ethics committee chair when she was told that her 46-year-old son David Christopher Dunn would have his LST removed in 10 days: "I said, 'Who gives you the right to kill my son? And he said, 'Oh Miss Kelly we don't call it killing—we call it comfort care'" (CBS News 2018). Jannette Nikolouzos said of her husband’s transfer to another hospital, "Spiro got out of that execution chamber that is St. Luke's" (Ackerman 2005). Her son told reporters that “a hospital shouldn't be able to tell you when it's going to euthanize your family members” (Ackerman 2005). Similarly, Mary Schindler said, “Euthanasia is rearing its ugly head out there right now” (Hannigan 2006). “Natural” death in the LCV worldview is not a death free of medical intervention, but a death that occurs because the heart gives out, rather than as a result of a human decision or action. Caterina Gonzalez succeeded in preventing the hospital from removing LST from 17-month-old Emilio, and when he died on a ventilator, she said he died "naturally, the way God intended" (Cohen 2007).

### The Timing of Death is God’s Decision

The timing of a human life’s death is a decision to be left in God’s hands, and any human action to determine the timing of someone’s death is hubris, i.e., “playing God.” Regarding the decision to remove her daughter’s feeding tube, Mary Schindler said, “Who are they to play God, anyway?” (Hannigan 2006). During her legal battle with her son’s hospital, Gonzalez told the press, “He may not live that long, but that’s nobody’s choice…that’s God’s choice” (NBC 2007). When he died, their attorney Jerri Ward said, "God chose to take Emilio at this time" (CBS NEWS 2007). In their fight to continue LST for Jahi McMath, her brother Omari Sealey said, “It’s not over until God say [sic] so” (Aviv 2018). Commenting on the TADA Hearings in 2006 (Hearings 2006), Colleen Horton, who was the public policy director of the Texas Center for Disability Studies at the University of Texas, said, "When did doctors, other medical professionals and lawyers become so infallible that we place decisions that we normally leave to God in their hands?" (Ackerman 2006).

While the foregoing depiction of the LCV hierarchy has been generated through anecdotal testimonials, there are compelling data to indicate that these tenets are shared by a substantial minority of the American population. For example, in the 2013 Pew Research Center’s project, “Views on End-of-Life Medical Treatment,” 31% of US adults said that “medical staff should do everything possible to save the life of the patient in all circumstances” (Pew 2013). When asked about their own end-of-life preferences, the percentages rose: 35% of adults said they would “tell their doctors ‘do everything possible to save their lives,’” even “if they had an incurable disease and were suffering a great deal of pain,” and 37% would want everything done even if “they were totally dependent on others with an incurable disease” (Pew 2013). The percentage of American adults who believe that life should be extended through medical intervention for as long as medically possible *doubled* between 1990 and 2013. The Pew data get even more granular, showing that the LCV worldview is the dominant value system in certain American subpopulations. For example, 61% of Black Protestants and 57% of Latinx Catholics say they would tell their doctors to do everything possible to save their lives even if they were experiencing a great deal of pain (Pew 2013). These data square with consistent evidence that both Black Americans (Johnson, Kuchibhatla & Tulsky 2008; Kwak & Haley 2005; True et al. 2005) and Latinx Americans (LoPresti, Dement & Gold 2016; Mack et al., 2010) want more aggressive LST than their white counterparts. Smith et al. reported that 45% of Black patients and 34% of the Latinx patients would want life-prolonging care even if they only had a few days to live (Smith et al. 2008). Data regarding the acceptance or rejection of “brain death” also ties the LCV worldview we depicted to empirical data. Siminoff et al. found that 30% of those surveyed in their study agreed with the claim, “a person is dead only when the heart has stopped beating” (Siminoff et al. 2003). When Sam Singer, the crisis communication expert brought in by the California hospital where Jahi McMath was declared brain-dead, said “She’s deceased by every law in the state of California. And by every spiritual belief system imaginable” (Aviv 2018), he was incorrect. Many evangelical Christians (Grisolia 2020), Native Americans and Muslims (Padela et al. 2013), and Orthodox Jews (Inwald et al. 2000; Verheijde et al. 2018; Segal 2014) do not recognize brain stem death as death of the individual. It is clear that many Americans subscribe to the LCV conception of the good, and it cannot be dismissed as a transitory phase or a fringe faction.

## Two Warring Conceptions of the Good, One Victor

I crafted these painstaking descriptions of the BIV and LCV conceptions of the good to demonstrate that they are both coherent, weighty, and widespread value systems. Neither the BIV nor the LCV is irrational, unintelligible, or normatively trivial. But in end-of-life and futility conflicts, the BIV hierarchy subjugates the LCV worldview. The BIV system has the monopoly on power in healthcare institutions, and the LCV adherents are a vulnerable minority who are much less likely to have their end-of-life treatment preferences honored for themselves or their loved ones. One study of older Americans near the end of life found that only 50% of patients who requested all possible interventions actually received values-concordant care (Silveira et al. 2010). A study of cancer patients found that only 9% of patients who preferred life extending treatment at the end of life received those interventions (Mack et al. 2010). It is not only the BIV-centric decisions of individual HCPs that constrain access to LST for LCV patients and surrogates. Most hospitals’ formal futility policies are thoroughly infused with BIV preferences and priorities. And while the BIV hierarchy dominates clinical practice and policy, it also dominates HEC. LCV adherents are not likely to have their positions bolstered by an ethics consultation if their dispute goes to the hospital’s ethics consult service. Hauschildt and DeVries’ 2020 study of HEC found that, in cases of disagreement between HCPs and SDMs, the ethics consultant typically deferred to the judgment of the HCPs and was not an independent voice. They concluded that “ethics consultations often reinforce the perspectives and preferences of clinicians” (Hauschildt and DeVries 2020, 317). The National Council on Disability’s 2019 report concurs with this conclusion: “Internal ethics committees are not an ideal forum for mediating and rendering medical futility decisions. By virtue of being a mechanism of the hospital, they are subject to financial, professional, and personal conflicts of interest,” that give “added weight, even unconsciously, to the opinions of the treating provider over the patient’s surrogate” (National Council on Disability 2019, 10) In the first 5 years of TADA, there were 47 futility disputes that went before an ethics committee for adjudication; the ethics consultants agreed with the clinical team 43 times (Fine and Mayo 2003). LCV patients and families are the weaker stakeholders in end-of-life and futility conflicts, with little recourse outside of legal action.

The current approach to futility disputes constitutes a hegemony of one American values system over another American values system. One conception of the good is being allowed to impose their healthcare values on individuals with radically different healthcare values. BIV adherents dictate the terms of end-of-life healthcare for LCV patients and SDMs because they are the numerical majority and hold the reins of power in healthcare decision-making. Yet many of the same BIV adherents are outraged when their healthcare values are profoundly violated, post-Roe, in states with anti-abortion majorities that prohibit them from performing or receiving an abortion. These are analogous instances of tyranny by the majority. In both cases, the implicit and explicit American covenant that citizens can live on their own terms and by their own values is being thwarted by the domination of one group’s value system. Futility policies will be viewed as trespassing on LCV adherents in exactly the same way that anti-abortion policies are viewed as trespassing on those who are pro-choice.

Up to this point in our argument, we have not addressed the looming objection that we are conflating LCV patients with LCV surrogates. On that objection, one might grant that BIV clinicians have an obligation to honor the known end-of-life preferences of LCV patients, but it does not follow that BIV clinicians have an obligation to honor the end-of-life choices of LCV surrogates when the patient’s own values are not known. My first rejoinder is that part of that objection is being driven by the moral certitude of the BIV adherents, who view themselves as knowing which clinical approach, goals of care, and treatment options are “best” for any patient at the end of life. And because they know what is in a patient’s best interest, they will naturally view the resistance or opposition of the patient’s family as evidence of a conflict of interest at best or malevolent motives at worst. But if we remove the presumption that HCPs inherently know what is in any patient’s best interest, then the claim that HCPs are better positioned than the patient’s family to determine what the patient would want or what would best serve the patient’s interests is a specious claim. My second rejoinder is to appeal to the empirical literature, which shows that the public wants their family to make end-of-life decisions and that SDMs have a high level of accuracy in predicting patients’ preferences (and much higher accuracy than HCPs). Pew found that 8 in 10 adults said that their closest family member should make decisions on their behalf at the end of life (Pew 2013). Although there are no data to substantiate this claim, I wonder whether the 20% who do not want family members to make decisions on their behalf have a much higher likelihood of having ACP documentation. Although the accuracy of surrogate decision-making is frequently quoted as a flip-of-the-coin 50%, SDMs have been shown to be 80% accurate when asked about treatment preferences for their loved one’s current health condition (Shalowitz 2006). Studies have also shown that hospital-based physicians are the worst predictors of patient preferences, ranking lower than primary care providers, and much lower than family surrogates (Coppola 2001). Empirical evidence contradicts the claim that HCPs know the values of their patients better than the patients’ SDMs. One recent study found that HCPs explored the patient’s values or religious beliefs in goals-of-care conversations fewer than 20% of the time (Ernecoff et al. 2015). When these data are combined with the evidence I provided earlier that LCV patients are less likely to have ACP documentation, there is a high likelihood that an LCV surrogate is accurately articulating the preferences of an LCV patient.

I have argued that a discernible, credible LCV worldview is frequently at the root of intractable futility conflicts with BIV stakeholders and that the power dynamics of BIV clinical care and policy result in values imposition on the less powerful LCV patients and families. I believe that such values imposition is not only ineffective in navigating end-of-life and futility disputes, but ethically suspect given the claims made by healthcare institutions that they do not discriminate based on creed, race, religion, or culture, all of which are contributing factors to the BIV and LCV worldviews. My conclusion is that HCPs (and clinical ethicists) have an obligation to engage much more seriously and respectfully with LCV patients and families. What would series engagement look like? Given the scope of this paper, we can only gesture here towards possible avenues to explore. The National Council on Disability has argued that futility decisions should be made by independent boards made up of individuals with a cross-section of values, members of the disability community, and HCPs and non-HCPs from diverse backgrounds (National Council on Disability 2019). They also advocate for a very limited scope for futility disputes, arguing “Because of these dangers, medical futility should only be defined in its limited, absolute sense. This narrow conception of futility is the closest to achieving objective, value-free decision making. All other definitions of futility almost invariably incorporate the value judgments of physicians” (2019, 35). Some groups are working on web-based tools for SDM’s to articulate patients’ values and priorities, which might make BIV HCPs more comfortable deferring to LCV surrogates (Suen 2020; Muehlschlegel et al. 2022). Perhaps tellingly, Muehlschlegel et al. found that SDMs using their tool to explore goals of care were 50% *less likely* to opt for comfort care measures over aggressive LST (Muehlschlegel et al. 2022). Another avenue would be to investigate the futility policies and procedures of institutions with a track record of LCV engagement. More than a decade after the implementation of Boston Children’s futility policy, the architects of the policy reported that they had not had *one* case that resulted in unilateral withdrawal of LST (Truog 2012).

The obvious objection against accommodation for families holding LCV conceptions of the good involves resource allocation. There are two versions of this objection; the first is financial, and the second focuses on scarcity. In the first version of the objection, an institution might argue that they simply cannot afford the expense of prolonging biological life when the treatment is no longer beneficial. On that objection, if “money were not an object,” then continuation of LST might be justified, but it is financially prohibitive for the institution to incur significant financial costs without offsetting medical benefit. The second version of the objection relates to the scarcity of ICU resources, claiming that those resources must be reserved for patients who can benefit by them and that it would be a moral failing of the institution to deny those resources to patients who could benefit because they are being used with patients who cannot receive any clinical benefit. My response to both of these objections is the same: if finances or scarce resources are the reason for limiting LST, that should be the justification given to the families for withdrawing LST. Institutions should be frank about these financial and moral pressures and not cloak their motivations by stating concerns about the patient’s “best interest.” There is a large minority of patients and families in the US who adhere to LCV, and if resource allocation is at the heart of LST usage and distribution, then that is a national conversation that we need to have because those problems have potential solutions (with a different type of commitment to medical funding, for example) and prioritizing those problems invite other serious bioethical problems (for example, conversations about limiting LST based on resource allocation reignites the debates about health equity from the 2020 dialogue about ventilator rationing).

What I have argued here is that incommensurable BIV and LCV conceptions of the good are firmly entrenched in American culture. I believe that the only way to stop the incessant surrogate wars is for BIV-focused healthcare institutions to engage more constructively with LCV stakeholders.

## References

[CR1] Abbott, K. H., Sago, J. G., Breen, C. M., Abernethy, A. P., & Tulsky, J. A. (2001). Families looking back: One year after discussion of withdrawal or withholding of life-sustaining support. *Critical Care Medicine,**29*(1), 197–201. 11176185 10.1097/00003246-200101000-00040

[CR2] Ackerman, T. (2005). Nursing home takes in man on ventilator, *Chron, *March 21. https://www.chron.com/news/houston-texas/article/Nursing-home-takes-in-man-on-ventilator-1679657.php

[CR3] Ackerman, T. (2006). Families urge change to state futile-care law. *Chron*, August 10. https://www.chron.com/news/houston-texas/article/families-urge-change-to-state-futile-care-law-1680292.php

[CR4] Aviv, R. (2018). What does it mean to die? *The New Yorker*, January 29. https://www.newyorker.com/magazine/2018/02/05/what-does-it-mean-to-die

[CR5] Azoulay, E., Timsit, J. F., Sprung, C. L., Soares, M., Rusinová, K., Lafabrie, A., Abizanda, R., et al. (2009). Prevalence and factors of intensive care unit conflicts: The conflicus study. *American Journal of Respiratory and Critical Care Medicine,**180*(9), 853–860. 10.1164/rccm.200810-1614OC 19644049 10.1164/rccm.200810-1614OC

[CR6] Barry, M. J. (1999). Involving patients in medical decisions: How can physicians do better? *JAMA,**282*(24), 2356–2357. 10612326 10.1001/jama.282.24.2356

[CR7] Bass, G. A., Chang, C. W. J., Winkle, J. M., Cecconi, M., Kudchadkar, S. R., Akuamoah-Boateng, K., Einav, S., Duffy, C. C., Hidalgo, J., Rodriquez-Vega, G. M., Gandra-d’Almeida, A. J., Barletta, J. F., & Kaplan, L. J. (2024). In-hospital violence and its impact on critical care practitioners. *Critical Care Medicine,**52*(7), 1113–1126. 38236075 10.1097/CCM.0000000000006189

[CR8] Batchelor, A., Jenal, L., Kapadia, F., Streat, S., Whetstine, L., & Woodcock, B. (2003). Ethics roundtable debate: Should a sedated dying patient be wakened to say goodbye to family? *Critical Care,**7*(5), 335–338. 10.1186/cc2329 12974961 10.1186/cc2329 PMC270714

[CR9] Beach, M. C., & Sugarman, J. (2019). Realizing shared decision-making in practice. *JAMA,**322*(9), 811–812. 31343669 10.1001/jama.2019.9797 PMC8786261

[CR10] Beauchamp, T. L., & Childress, J. F. (1979). *Principles of biomedical ethics*. Oxford University Press.

[CR11] Berger, S., Grzonka, P., Frei, A. I., Hunziker, S., Baumann, S. M., Amacher, S. A., Gebhard, C. E., & Sutter, R. (2024). Violence against healthcare professionals in intensive care units: A systematic review and meta-analysis of frequency, risk factors, interventions, and preventive measures. *Critical Care,**28*(1), 61. 38409034 10.1186/s13054-024-04844-z PMC10898135

[CR12] Boggs, K. (2006). FIRST-PERSON: Passive euthanasia in America, *Baptist Press*, April 28. https://www.baptistpress.com/resource-library/news/first-person-passive-euthanasia-in-america/

[CR13] Bosslet, G. T., Pope, T. M., Rubenfeld, G. D., Lo, B., Truog, R. D., Rushton, C. H., Randall Curtis, J., et al. (2015). An official ATS/AACN/ACCP/ESICM/SCCM policy statement: Responding to requests for potentially inappropriate treatments in intensive care units. *American Journal of Respiratory and Critical Care Medicine,**191*(11), 1318–1330. 10.1164/rccm.201505-0924ST 25978438 10.1164/rccm.201505-0924ST

[CR14] Breen, C. M., Abernethy, A. P., Abbott, K. H., & Tulsky, J. A. (2001). Conflict associated with decisions to limit life-sustaining treatment in intensive care units. *Journal of General Internal Medicine,**16*(5), 283–289. 11359545 10.1046/j.1525-1497.2001.00419.x PMC1495214

[CR15] CBS News (2007). Sick infant in life support debate dies. May 21. https://www.cbsnews.com/news/sick-infant-in-life-support-debate-dies/

[CR16] CBS Texas Staff (2018). Texas mom wants to change way medical decisions are made after losing son-CBS Texas, October 25. https://www.cbsnews.com/texas/news/texas-mom-medical-decisions-losing-son/

[CR17] CBS Texas Staff (2022). Tinslee Lewis discharged from Cook Children's Medical Center amid legal fight. April 13. https://www.cbsnews.com/texas/news/tinslee-lewis-discharged-cook-childrens-medical-center-legal-fight/

[CR18] Childress, J. F., & Childress, M. D. (2020). What does the evolution from informed consent to shared decision making teach us about authority in health care? *AMA Journal of Ethics,**22*(5), E423-429. 32449659 10.1001/amajethics.2020.423

[CR19] Chow, K. (2018) Jahi McMath, teen at center of medical and religious debate on brain death, has died. *NPR*, June 29, 2018, sec. News. https://www.npr.org/2018/06/29/624641317/jahi-mcmath-teen-at-center-of-medical-and-religious-debate-on-brain-death-has-di

[CR20] Cohen, E. (2007). Fight over baby’s life support divides ethicists - CNN.com. April 25, 2007. https://www.cnn.com/2007/HEALTH/04/25/baby.emilio/index.html

[CR21] Coppola, K. M., Ditto, P. H., Danks, J. H., & Smucker, W. D. (2001). Accuracy of primary care and hospital-based physicians’ predictions of elderly outpatients’ treatment preferences with and without advance directives. *Archives of Internal Medicine,**161*(3), 431–440. 10.1001/archinte.161.3.431 11176769 10.1001/archinte.161.3.431

[CR22] Entwistle, V. A., & Watt, I. S. (2016). Broad versus narrow shared decision making: Patients’ involvement in real world contexts. In G. Elwyn, A. Edwards, & R. Thompson (Eds.), *Shared decision making in health care: Achieving evidence-based patient choice, *third edition (7–12). Oxford University Press.

[CR23] Epstein, E. G., Whitehead, P. B., Prompahakul, C., Thacker, L. R., & Hamric, A. B. (2019). Enhancing understanding of moral distress: The measure of moral distress for health care professionals. *AJOB Empirical Bioethics,**10*(2), 113–124. 10.1080/23294515.2019.1586008 31002584 10.1080/23294515.2019.1586008

[CR24] Ernecoff, N. C., Curlin, F. A., Buddadhumaruk, P., & White, D. B. (2015). Health care professionals’ responses to religious or spiritual statements by surrogate decision makers during goals-of-care discussions. *JAMA Internal Medicine,**175*(10), 1662–1669. 10.1001/jamainternmed.2015.4124 26322823 10.1001/jamainternmed.2015.4124

[CR25] Feudtner, C., & Nathanson, P. G. (2018). Futility, inappropriateness, conflict, and the complexity of medical decision-making. *Perspectives in Biology and Medicine,**60*(3), 345–357. 10.1353/pbm.2018.0008 29375064 10.1353/pbm.2018.0008

[CR26] Fiester, A. (2014). When it hurts to ask: Avoiding moral injury in requests to forgo treatment. *American Journal of Physical Medicine & Rehabilitation,**93*(3), 260–262. 10.1097/PHM.0b013e3182a51e0a 24561319 10.1097/PHM.0b013e3182a51e0a

[CR27] Fine, R. L. (2000). Medical futility and the Texas Advance Directives Act of 1999. *Baylor University Medical Center,**13*(2), 144–147. 10.1080/08998280.2000.11927658 PMC1312296 16389368

[CR28] Fine, R. L. (2009). Point: The Texas Advance Directives Act effectively and ethically resolves disputes about medical futility. *Chest,**136*(4), 963–967. 10.1378/chest.09-1267 19809044 10.1378/chest.09-1267

[CR29] Fromson, N. (2022). “My Life Matters”: Resilience after traumatic spinal cord injury. May 20, 2022. https://www.michiganmedicine.org/health-lab/my-life-matters-resilience-after-traumatic-spinal-cord-injury

[CR30] Frosch, D. L., May, S. G., Rendle, K. A., et al. (2012). Authoritarian physicians and patients’ fear of being labeled “difficult” among key obstacles to shared decision making. *Health Aff Millwood.,**31*(5), 1030–1038. 22566443 10.1377/hlthaff.2011.0576

[CR31] Garrido, M. M., Idler, E. L., Leventhal, H., & Carr, D. (2013). Pathways from religion to advance care planning: Beliefs about control over length of life and end-of-life values. *The Gerontologist,**53*(5), 801–816. 10.1093/geront/gns128 23161430 10.1093/geront/gns128 PMC3771671

[CR32] Goldschmidt, D. (2018). Jahi McMath, California teen at center of brain-death controversy, has died. CNN, June 29, https://www.cnn.com/2018/06/29/health/jahi-mcmath-brain-dead-teen-death/index.html

[CR33] Grisolia, J. S. (2020). The world brain death project: Answering the wrong questions. August 30, https://www.medpagetoday.com/publichealthpolicy/healthpolicy/88327

[CR34] Hannigan, J. (2006). Terri Schiavo’s parents reflect on the year since her death. *Baptist Press*. April 11. https://www.baptistpress.com/resource-library/news/terri-schiavos-parents-reflect-on-the-year-since-her-death/

[CR35] Hauschildt, K. E. (2022). Whose good death? Valuation and standardization as mechanisms of inequality in hospitals. *Journal of Health and Social Behavior*. 10.1177/00221465221143088 10.1177/00221465221143088 PMC10267289 36523154

[CR36] Hauschildt, K., & De Vries, R. (2020). Reinforcing medical authority: Clinical ethics consultation and the resolution of conflicts in treatment decisions. *Sociology of Health & Illness,**42*(2), 307–326. 10.1111/1467-9566.13003 31565808 10.1111/1467-9566.13003 PMC7012693

[CR37] Hearings before the house committee on public health. (2006). https://senate.texas.gov/cmte-arch.php?c=610&s=79. Accessed 15 Nov.2024

[CR38] Houston Chronicle (2007). Edith Pereira obituary - Houston, TX. Legacy.com. Accessed August 30, 2023. https://www.legacy.com/us/obituaries/houstonchronicle/name/edith-pereira-obituary?id=26479772

[CR39] Iezzoni, L. I., Rao, S. R., Ressalam, J., Bolcic-Jankovic, D., Agaronnik, N. D., Donelan, K., Lagu, T., & Campbell, E. G. (2021). Physicians’ perceptions of people with disability and their health care. *Health Affairs (Project Hope),**40*(2), 297–306. 10.1377/hlthaff.2020.01452 33523739 10.1377/hlthaff.2020.01452 PMC8722582

[CR40] Inwald, D., Jakobovits, I., & Petros, A. (2000). Brain stem death: Managing care when accepted medical guidelines and religious beliefs are in conflict. Consideration and compromise are possible. *BMJ (Clinical Research Ed.),**320*(7244), 1266–1267. 10.1136/bmj.320.7244.1266 10.1136/bmj.320.7244.1266 PMC1117998 10797044

[CR41] Janvier, A., Farlow, B., & Wilfond, B. S. (2012). The experience of families with children with trisomy 13 and 18 in social networks. *Pediatrics,**130*(2), 293–298. 10.1542/peds.2012-0151 22826570 10.1542/peds.2012-0151

[CR42] Johnson, K. S., Kuchibhatla, M., & Tulsky, J. A. (2008). What explains racial differences in the use of advance directives and attitudes toward hospice care? *Journal of the American Geriatrics Society,**56*(10), 1953–1958. 10.1111/j.1532-5415.2008.01919.x 18771455 10.1111/j.1532-5415.2008.01919.x PMC2631440

[CR43] Kaps, B., Alexander Chen, H., Kopf, G. S., & Encandela, J. (2021). Perspectives on the effectiveness of a medical futility policy. *The Journal of Clinical Ethics,**32*(1), 48–60. 33656456

[CR500] Kingsley, J., Clark, J., Lewis-Newby, M., Dudzinski, D.M., & Diekema, D. (2023). Navigating parental requests: Considering the relational potential standard in paediatric end-of-life care in the paediatric intensive care unit.* Journal of Medical Ethics,* 15. 10.1136/jme-2023-108912 10.1136/jme-2023-108912 37968108

[CR44] Kopelman, L. M. (1997). The best-interests standard as threshold, ideal, and standard of reasonableness. *The Journal of Medicine and Philosophy,**22*(3), 271–289. 10.1093/jmp/22.3.271 9232512 10.1093/jmp/22.3.271

[CR45] Kwak, J., & Haley, W. E. (2005). Current research findings on end-of-life decision making among racially or ethnically diverse groups. *The Gerontologist,**45*(5), 634–641. 10.1093/geront/45.5.634 16199398 10.1093/geront/45.5.634

[CR46] Leland, B. D., Torke, A. M., Wocial, L. D., & Helft, P. R. (2017). Futility disputes: A review of the literature and proposed model for dispute navigation through trust building. *Journal of Intensive Care Medicine,**32*(9), 523–527. 10.1177/0885066616666001 27568477 10.1177/0885066616666001

[CR47] Liou, T. (2022). Mom, “filled with joy,” announces daughter Tinslee Lewis is home after years-long legal fight to keep her on life support. wfaa.com, April 4. https://www.wfaa.com/article/news/local/fort-worth-child-tinslee-lewis-now-home-after-fight-to-keep-her-on-life-support-mom-says/287-61a13670-0b0e-4c7b-97a7-527c8c918c0a

[CR48] Long, A. C., & Randall Curtis, J. (2014). The epidemic of physician-family conflict in the ICU and what we should do about it. *Critical Care Medicine,**42*(2), 461–462. 10.1097/CCM.0b013e3182a525b8 24434450 10.1097/CCM.0b013e3182a525b8

[CR49] LoPresti, M. A., Dement, F., & Gold, H. T. (2016). End-of-life care for people with cancer from ethnic minority groups: A systematic review. *The American Journal of Hospice & Palliative Care,**33*(3), 291–305. 10.1177/1049909114565658 25550406 10.1177/1049909114565658

[CR50] Mack, J. W., Elizabeth Paulk, M., Viswanath, K., & Prigerson, H. G. (2010). Racial disparities in the outcomes of communication on medical care received near death. *Archives of Internal Medicine,**170*(17), 1533–1540. 10.1001/archinternmed.2010.322 20876403 10.1001/archinternmed.2010.322 PMC3739279

[CR51] Malek, J., & Nelson, R. (2022). Should he have brain surgery? *The Hastings Center*, November 29. https://www.thehastingscenter.org/should-he-have-brain-surgery/

[CR52] Morley, G., & Sankary, L. R. (2023). Re-examining the relationship between moral distress and moral agency in nursing. *Nursing Philosophy*. 10.1111/nup.12419 10.1111/nup.12419 36748963

[CR53] Morrison, R. S., Meier, D. E., & Arnold, R. M. (2021). What’s wrong with advance care planning. *JAMA,**326*(16), 1575–1576. 10.1001/jama.2021.16430 34623373 10.1001/jama.2021.16430 PMC9373875

[CR54] Muehlschlegel, S., Goostrey, K., Flahive, J., Zhang, Q., Pach, J. J., & Hwang, D. Y. (2022). A pilot randomized clinical trial of a goals-of-care decision aid for surrogates of severe acute brain injury patients. *Neurology,**99*(14), e1446-1455. 10.1212/WNL.0000000000200937 35853748 10.1212/WNL.0000000000200937 PMC9576301

[CR55] Nathanson, P., & Feudtner, C. (2016). Futility and the alluring fantasy of avoiding conflict. *A & A Case Reports,**6*(7), 186–187. 27035369 10.1213/XAA.0000000000000224

[CR56] National Council on Disability. (2019). Medical futility and disability bias: Part of the bioethics and disability series, November 20.

[CR57] NBC News. (2007). Family, hospital clash on life-support for baby. April 10. http://www.nbcnews.com/health/health-news/family-hospital-clash-life-support-baby-flna1C9474665

[CR58] Padela, A. I., Arozullah, A., & Moosa, E. (2013). Brain death in Islamic ethico-legal deliberation: Challenges for applied Islamic bioethics. *Bioethics,**27*(3), 132–139. 10.1111/j.1467-8519.2011.01935.x 22150919 10.1111/j.1467-8519.2011.01935.x

[CR59] Peace, W. J. (2012). Comfort care as denial of personhood. *The Hastings Center Report,**42*(4), 14–17. 10.1002/hast.38 22777973

[CR60] Pew Research Center. (2013). Views on end-of-life medical treatments. *Pew Research Center’s Religion & Public Life Project* (blog), November 21. https://www.pewresearch.org/religion/2013/11/21/views-on-end-of-life-medical-treatments/

[CR61] Pope, T. (2016). Texas Advance Directives Act: Nearly a model dispute resolution mechanism for intractable medical futility conflicts. Faculty Scholarship, January 1. https://open.mitchellhamline.edu/facsch/378.

[CR62] Potter, J., & Lee, S. W. (2020). When advance directives collide. *Journal of General Internal Medicine,**35*(7), 2191–2192. 10.1007/s11606-020-05680-x 32020492 10.1007/s11606-020-05680-x PMC7352021

[CR63] Prigerson, H. G., Viola, M., Maciejewski, P. K., & Falzarano, F. (2023). Advance care planning (ACP) to promote receipt of value-concordant care: Results vary according to patient priorities. *PLoS ONE,**18*(1), e0280197. 10.1371/journal.pone.0280197 36630471 10.1371/journal.pone.0280197 PMC9833543

[CR64] Rawls, J. (1999). *A theory of justice* (Revised). Belknap Press.

[CR65] Rosoff, P. M. (2013). Institutional futility policies are inherently unfair. *HEC Forum: An interdisciplinary* *Journal on Hospitals’ Ethical and Legal Issues,**25*(3), 191–209. 10.1007/s10730-012-9194-9 10.1007/s10730-012-9194-9 22903421

[CR66] Salter, E. K. (2012). Deciding for a child: A comprehensive analysis of the best interest standard. *Theoretical Medicine and Bioethics,**33*(3), 179–198. 10.1007/s11017-012-9219-z 22528148 10.1007/s11017-012-9219-z

[CR67] Schindler, M., Schindler, R. (2006). *A life that matters: The legacy of Terri Schiavo--A lesson for us all*. First Edition. Grand Central Publishing.

[CR68] Schmid, B., Allen, R. S., Haley, P. P., & Decoster, J. (2010). Family matters: Dyadic agreement in end-of-life medical decision making. *The Gerontologist,**50*(2), 226–237. 10.1093/geront/gnp166 20038541 10.1093/geront/gnp166 PMC2838411

[CR69] Schuster, R. A., Hong, S. Y., Arnold, R. M., & White, D. B. (2014). Investigating conflict in ICUs - Is the clinicians’ perspective enough? *Critical Care Medicine,**42*(2), 328–335. 10.1097/CCM.0b013e3182a27598.PMID:24434440;PMCID:PMC3902111 24434440 10.1097/CCM.0b013e3182a27598 PMC3902111

[CR70] Segal, E. (2014). Religious objections to brain death. *Journal of Critical Care,**29*(5), 875–877. 10.1016/j.jcrc.2014.06.017 25085509 10.1016/j.jcrc.2014.06.017

[CR71] Shalowitz, D. I., Garrett-Mayer, E., & Wendler, D. (2006). The accuracy of surrogate decision makers: A systematic review. *Archives of Internal Medicine,**166*(5), 493–497. 10.1001/archinte.166.5.493 16534034 10.1001/archinte.166.5.493

[CR72] Shea, M. (2020). Principlism’s balancing act: Why the principles of biomedical ethics need a theory of the good. *The Journal of Medicine and Philosophy,**45*(4–5), 441–470. 10.1093/jmp/jhaa014 32726809 10.1093/jmp/jhaa014

[CR73] Silveira, M. J., Kim, S. Y. H., & Langa, K. M. (2010). Advance directives and outcomes of surrogate decision making before death. *The New England Journal of Medicine,**362*(13), 1211–1218. 10.1056/NEJMsa0907901 20357283 10.1056/NEJMsa0907901 PMC2880881

[CR74] Siminoff, L. A., Mercer, M. B., & Arnold, R. (2003). Families’ understanding of brain death. *Progress in Transplantation,**13*(3), 218–224. 10.1177/152692480301300309 14558637 10.1177/152692480301300309

[CR75] Sjöberg, F., Salzmann-Erikson, M., Åkerman, E., Joelsson-Alm, E., & Schandl, A. (2024). The paradox of workplace violence in the intensive care unit: A focus group study. *Critical Care,**28*(1), 232. 38992709 10.1186/s13054-024-05028-5 PMC11241930

[CR76] Slack, R. J., French, C., McGain, F., Bates, S., Gao, A., Knowles, S., & Yang, Y. (2023). George institute for global health and the australian and new zealand intensive care society clinical trials group (ANZICS CTG). violence in intensive care: a point prevalence study. *Critical Care and Resuscitation,**24*(3), 272–279. 38046215 10.51893/2022.3.OA7 PMC10692600

[CR77] Smith, A. K., Lo, B., & Sudore, R. (2013). When previously expressed wishes conflict with best interests. *JAMA Internal Medicine,**173*(13), 1241–1245. 10.1001/jamainternmed.2013.6053 23712743 10.1001/jamainternmed.2013.6053 PMC3741042

[CR78] Smith, A. K., McCarthy, E. P., Paulk, E., Balboni, T. A., Maciejewski, P. K., Block, S. D., & Prigerson, H. G. (2008). Racial and ethnic differences in advance care planning among patients with cancer: Impact of terminal illness acknowledgment, religiousness, and treatment preferences. *Journal of Clinical Oncology,**26*(25), 4131–4137. 10.1200/JCO.2007.14.8452 18757326 10.1200/JCO.2007.14.8452 PMC2654372

[CR79] Studdert, D. M., Mello, M. M., Burns, J. P., Puopolo, A. L., Galper, B. Z., Truog, R. D., & Brennan, T. A. (2003). Conflict in the care of patients with prolonged stay in the ICU: Types, sources, and predictors. *Intensive Care Medicine,**29*(9), 1489–1497. 12879243 10.1007/s00134-003-1853-5

[CR80] Suen, A. O., Butler, R. A., Arnold, R., Myers, B., Witteman, H. O., Cox, C. E., Argenas, A., et al. (2020). Developing the family support tool: An interactive, web-based tool to help families navigate the complexities of surrogate decision making in ICUs. *Journal of Critical Care,**56*, 132–139. 10.1016/j.jcrc.2019.12.002 31896447 10.1016/j.jcrc.2019.12.002 PMC7646226

[CR81] Texas Law House Bill (HB) 3162 (2023). https://capitol.texas.gov/tlodocs/88R/billtext/html/HB03162I.htm

[CR82] Traudt, T., Liaschenko, J., & Peden-McAlpine, C. (2016). Moral agency, moral imagination, and moral community: Antidotes to moral distress. *The Journal of Clinical Ethics,**27*(3), 201–213. 27658275

[CR83] True, G., Phipps, E. J., Braitman, L. E., Harralson, T., Harris, D., & Tester, W. (2005). Treatment preferences and advance care planning at end of life: The role of ethnicity and spiritual coping in cancer patients. *Annals of Behavioral Medicine: A Publication of the Society of Behavioral Medicine,**30*(2), 174–179. 10.1207/s15324796abm3002_10 16173914 10.1207/s15324796abm3002_10

[CR84] Truog, R. (2012). Medical futility. *Georgia State University Law Review,**25*(4), 985–1002. https://readingroom.law.gsu.edu/gsulr/vol25/iss4/13

[CR85] Truog, R. D., & Mitchell, C. (2006). Futility–From hospital policies to state laws. *The American Journal of Bioethics, **6*(5), 19–21. 10.1080/15265160600858989. 10.1080/15265160600858989 16997814

[CR86] Unger, T. (2018). Give her a chance: North Texas family fights to keep 9-year-old on life support.” wfaa.com, September 8. https://www.wfaa.com/article/news/local/tarrant-county/give-her-a-chance-north-texas-family-fights-to-keep-9-year-old-on-life-support/287-599611360

[CR87] Veatch, R. M. (1995). Abandoning informed consent. *The Hastings Center Report,**25*(2), 5–12. 10.2307/3562859 7782200

[CR88] Verheijde, J. L., Rady, M. Y., & Potts, M. (2018). Neuroscience and brain death controversies: The elephant in the room. *Journal of Religion and Health,**57*(5), 1745–1763. 10.1007/s10943-018-0654-7 29931477 10.1007/s10943-018-0654-7 PMC6132575

[CR89] Wfaa.com (2019). Temporary restraining order prevents hospital from taking 9-month-old girl off life support. November 11. https://www.wfaa.com/article/news/health/9-month-old-baby-to-be-taken-off-life-support-sunday-against-mothers-wishes-advocacy-group-says/287-40975c35-8ce5-4f35-8441-2aa9bf21b66d

[CR90] Wicclair, M. R., & White, D. B. (2014). Surgeons, intensivists, and discretion to refuse requested treatments. *The Hastings Center Report,**44*(5), 33–42. 10.1002/hast.356 10.1002/hast.356 25231660

[CR91] Wilson, M. E., Dobler, C. C., Zubek, L., Gajic, O., Daniel Talmor, J., Curtis, R., Hinds, R. F., et al. (2019). Prevalence of disagreement about appropriateness of treatment between ICU patients/surrogates and clinicians. *Chest,**155*(6), 1140–1147. 10.1016/j.chest.2019.02.404 30922949 10.1016/j.chest.2019.02.404

